# The use of intralipid emulsion therapy to treat severe cardiotoxicity secondary to lamotrigine ingestion in a dog

**DOI:** 10.1002/ccr3.1733

**Published:** 2018-08-30

**Authors:** Tara J. Bellis, Laura Gibeon

**Affiliations:** ^1^ Emergency and Critical Care Department BluePearl Veterinary Partners New York New York

**Keywords:** intralipid, lamotrigine, tachyarrhythmia, toxicity

## Abstract

Lamotrigine is a sodium and calcium channel blocker, used to treat seizures in people. Dogs metabolize Lamotrigine to a cardiotoxic metabolite that causes severe, often fatal ventricular arrhythmias. This report documents the successful treatment of refractory Lamotrigine cardiotoxicity in a dog, using intralipid emulsion therapy.

## 1 INTRODUCTION

Lamotrigine (LTG) is a phenyltriazine anticonvulsant medication used in people to treat seizures and bipolar disorders.^1^ Lamotrigine blocks voltage‐sensitive sodium channels and high‐voltage activated calcium‐channels, reduces neuronal glutamate release and inhibits serotonin reuptake by weakly inhibiting 5HT3.^1^ Lamotrigine may also have potassium channel blocking actions.^2^ Peak concentrations in the blood are reached within 1‐3 hours following ingestion, and 12 hours with the extended‐release formulation in both people and animals, and the half‐life is 24‐30 hours.^1^ LTG is predominantly metabolized by the liver and undergoes hepatic glucuronidation before elimination by the kidneys in people.^1^ In dogs, LTG is metabolized in the liver to a toxic metabolite; LTG‐2‐N‐methyl, by a species‐specific methyltransferase, prior to elimination by the kidneys. This metabolite causes severe cardiac disturbances in a dose‐dependent manner; prolongation of the PR interval, widening of the QRS complex, and, at higher doses, complete AV conduction block. Only trace amounts of the 2‐N‐methyl metabolite have been documented in human urine.^3^ Lamotrigine is generally well tolerated in people. A hypersensitivity reaction can occur in the form of a rash, which can be severe. Cardiac and central nervous system (CNS) effects, as well as serotonin‐like syndromes have been reported. Seizure aggravation can occur in people with pre‐existing seizure disorders.^4^ Clinical signs of toxicity include lethargy, drowsiness, nausea, vomiting, confusion, agitation, ataxia, tachycardia, nystagmus, blurred vision, coma, and death.^4^ Treatment options for arrhythmias induced by LTG include administration of lidocaine, and alkalization via administration of sodium bicarbonate (NaHCO_3_). Due to the lipid solubility of LTG^2^ (logP 2,4), intralipid emulsion therapy (ILE) has been considered as a potential treatment and is recommended in both people and animals when cardiotoxicity is refractory to treatment.^1^, ^2^, ^5^, ^6^, ^7^, ^8^, ^9^ Recommendations for veterinary patients are extrapolated from the human literature, and anecdotal reports from the American Society for the Protection of Animals Animal Poison Control Center (ASPCA APCC). This is the first case report, to the authors' knowledge, describing the successful use of ILE for the treatment of severe cardiotoxicity secondary to LTG ingestion in a dog.

## 2 CASE SUMMARY

A 2‐year‐old female spayed English Bulldog, weighing 23.1 kg, presented to the authors hospital following accidental ingestion of 600 mg of LTG (26 mg/kg) up to ten hours prior. Six hours before presentation, the dog vomited multiple times, and was noted by the owner to be severely lethargic. Medical history was otherwise unremarkable. Upon presentation (day one), the patient was mentally dull and laterally recumbent. Pupillary light reflexes and menace responses were decreased bilaterally. A comprehensive neurologic examination was not performed. Heart rate was 210 beats per minute (bpm) with an irregular rhythm. Respiratory rate was 42 breaths per minute. Oxygen saturation as obtained via pulse oximetry (Pulse oximeter; Masimo Corporation, Irvine, CA) ranged from 96%‐100%. Flow by oxygen was provided at 2 L/min during the stabilization period. Thoracic auscultation was within normal limits. Ptyalism was noted. The remainder of the physical examination was unremarkable. Systolic blood pressure (SBP) was unreadable via Doppler ultrasonography (Ultrasonic Doppler Flow Detector; Parks Medical Electronics Inc., Aloha, OR). The patient had an ECG placed, and ventricular tachycardia (VT) was observed. Packed cell volume (PCV) was 74% (range 35%‐45%), total protein (TP) was 8.0 g/dL (range 6.5‐8 g/dL), and blood glucose was 100 mg/dL (range 70‐130 mg/dL). Venous blood gas (VBG; Siemans Healthcare Diagnostics Inc., Tarrytown, NY) analysis revealed a metabolic acidosis and hyperlactatemia at 8.85 mmol/L (range 1‐2 mmol/L; Table 1).

**Table 1:** Venous blood gas and electrolyte analyses in a dog presented for lamotrigine toxicity

Parameter	Day 1 am	Day 1 pm	Day 2 am	Day 2 pm	Day 3 am	Reference interval
pH	7.218	7.320	7.387	7.41	7.397	7.3‐7.37
pCO_2_ (mm Hg)	48.3	36.7	43	42.9	39	28‐46
HCO_3_ (mmol/L)	19.2	18.5	25.3	26.6	23.5	19.4 ± 4
BE (mmol/L)	−8.8	−6.4	0	1.9	−1.1	−4‐+4
Na+ (mmol/L)	151.5	145	142.9	143.8	141.3	146‐156
K+ (mmol/L)	3.46	3.76	4.17	4.22	4.35	3.5‐5.5
Ca++ (mmol/L)	1.3	1.24	1.23	1.12	1.07	1.2‐1.5
Cl (mmol/L)	109	110	109	108	109	109‐122
Glucose (mg/dL)	100	68	138	122	110	79‐126
Lactate(mmol/L)	8.85	6.21	1.77	1.09	0.85	1‐2

A 20‐Gauge IV catheter (Insyte catheter; Becton Dickinson Infusion Therapy Systems Inc., Sandy, UT) was placed in the left cephalic vein, and IV crystalloid fluid therapy was initiated (80 mL/h LRS (Lactated Ringers injection; Dechra Veterinary Products, Overland Park, KS) supplemented with 20 meq of KCl (Potassium Chloride injection 2 meq/mL, Hospira Inc Lake Forest, IL)/L). A lidocaine (Lidocaine Hydrochloride Injectable 2% Phoenix TM; Manufactured for Clipper Distributing Company, LLC St. Joseph, MO or Sparhawk Laboratories Inc., Lenexa, KS) bolus of 2 mg/kg IV was administered, and the VT temporarily converted to normal sinus rhythm (NSR). Two further lidocaine (Manufactured for Clipper Distributing Company, LLC or Sparhawk Laboratories Inc.) boluses were administered (2 mg/kg IV) prior to starting a continuous rate infusion (CRI) of lidocaine (Manufactured for Clipper Distributing Company, LLC or Sparhawk Laboratories Inc.) at 50 mcg/kg/min IV. The heart rate initially slowed to 140 bpm; however, the VT re‐occurred at a rate of 200 bpm, and the CRI of lidocaine (Manufactured for Clipper Distributing Company, LLC or Sparhawk Laboratories Inc.) was increased to 75 mcg/kg/min IV. Additional treatments included maropitant citrate (Maropitant citrate 10 mg/mL; Zoetis Inc., Kalamazoo, MI; 1 mg/kg IV q24), pantoprazole (Pantoprazole 40 mg per vial; AuroMedics Pharma LLC, East Windsor, NJ; 1 mg/kg IV SID), and metoclopramide (Metoclopramide Hydrochloride, Hospira Inc, Lake Forest, IL; 1 mg/kg IV, followed by a CRI at 1 mg/kg/day).

The patient remained mentally dull. Recheck PCV and TP were 65% (range 35%‐45%) and 4.0 g/dL (range 6.5‐8 g/dL), respectively. Repeat VBG (Siemans Healthcare Diagnostics Inc.) analysis revealed a mild improvement in the metabolic acidosis, and persistence of the hyperlactatemia (Table 1). The patient developed mild hypoglycemia; BG 68 g/dL (range 70‐130 mg/dL), and 2.5% dextrose (50% dextrose injection, USP 25 g/50 mL (0.5 g/mL) Hospira Inc., Lake Forest, IL) supplementation was added to the IVF. Due to the concern that the hypoglycemia could be secondary to evolving aspiration pneumonia, antibiotic therapy was initiated in the form of enrofloxacin (Enrofloxacin 2.27% Bayer HealthCare LLC, Shawnee, KS; 15 mg/kg IV SID), and unasyn (Ampicillin and Sulbactam for injection 1.5 g/vial, MITIM S.R.L, Brescia, Italy; 50 mg/kg IV TID). Systolic blood pressure was obtained via Doppler (Parks Medical Electronics Inc.), and was low at 40 mm Hg (range 100‐160 mm Hg). Two 20 mL/kg IV boluses of LRS (Dechra Veterinary Products) and one 10 mL/kg IV bolus of LRS were administered and the SBP improved to 80 mm Hg (range 100‐160 mm Hg).

Telemetry (hts 820TC Trenscenter Central Station Cardiac Monitor, DRE Veterinary, Louisville, KY, USA) was placed and persistent VT was observed at a rate of 210 bpm. The patient had severe pulse deficits (PR 40 bpm), and intermittent periods of bradycardia (HR 40‐60 bpm), at times consistent with AV block. Sodium bicarbonate (Sodium bicarbonate 8.4%; Nova‐Tech, Grand Island, NE; 2 × 0.5 mEq/kg slow IV bolus, given 30 min apart) was administered for alkalization. The rhythm intermittently revealed a sine wave, indicative of potential ventricular fibrillation. At this time the lidocaine CRI was discontinued, and magnesium sulfate (Magnesium sulphate 50%; XGen, Big Flats, NY; 0.15 mcg/kg IV) was administered for cardioprotection. No improvement to the rhythm was appreciated, and the patient was minimally responsive; therefore intralipid emulsion (ILE, Intralipid 20%; Baxter Healthcare Corporation, Deerfield, IL) 2 mL/kg was administered as an IV bolus, and then started as a CRI (0.25 mL/kg/min IV). Normal sinus rhythm was observed upon initiation of ILE. Intermittent re‐occurrence of the VT was noted, and three further boluses of lidocaine (Manufactured for Clipper Distributing Company, LLC or Sparhawk Laboratories Inc.; 2 mg/kg IV) were administered, resulting in conversion to NSR each time. Systolic blood pressure remained low, ranging between 74‐88 mm Hg (100‐160 mm Hg), and a CRI of norepinephrine (Levophed™ (Norepinephrine Bitartrate Injection, USP) Hospira Inc., Lake Forest, IL) was started at 0.1 mcg/kg/min IV and titrated up to 0.3 mcg/kg/min IV.

Blood was collected for a complete blood count (CBC; Idexx Reference Laboratory, New York, NY, USA) and serum biochemical profile (Idexx Reference Laboratory) (Tables 2 and 3). The CBC revealed polycythemia, and a mild inflammatory leukogram. The biochemistry panel revealed panhypoproteinemia, increased ALT, AST and total bilirubin, and decreased cholesterol. Hypertriglyceridemia was noted, likely secondary to the administration of ILE (Table 3). Three‐view thoracic radiographs were obtained and revealed a patchy area of alveolar pattern overlying the left cranioventral lung lobe, most compatible with atelectasis or early aspiration pneumonia. A mild bronchointerstitial pattern was noted in the remaining pulmonary parenchyma. Approximately 6 hours following initiation of ILE therapy, blood pressure had normalized (SBP 110 mm Hg; range 100‐160 mm Hg), and the norepinephrine (Levophed™) CRI was weaned and discontinued. Continued monitoring of VBGs demonstrated improvements in the metabolic acidosis and resolution of the hyperlactatemia (Table 1). The patient became more neurologically appropriate and ambulatory. The ECG revealed NSR with an occasional idioventricular rhythm.

**Table 2:** Complete blood count values in a dog presented for lamotrigine toxicity

Parameter	Concentration Day 1	Concentration Day 2	Reference interval
Hematocrit (%)	61	57	38.3‐56.5
Hemoglobin (g/dL)	24.7	20.9	13.4‐20.7
MCV (fl)	76	74	59‐76
MCH (pg)	30.9	27	21.9‐26.1
MCHC (g/dL)	40.5	36.7	32.6‐39.2
Reticulocytes (%)	0.9	0.7	n/a
Reticulocyte (K/μL)	72	54	10‐110
WBC (K/μL)	22	20.5	4.9‐17.6
Neutrophil (K/μL)	19.58	19.27	2.94‐12.67
Band (K/μL)	220	410	0‐170
Lymphocytes (K/μL)	1.1	0.41	1.06‐4.95
Monocytes (K/μL)	0.88	0.41	0.13‐1.15
Eosinophils (K/μL)	0.22	0	0.07‐1.49
Basophils (K/μL)	0	0	0‐0.1
Platelet (K/μL)	Adequate*	190	143‐448

*Automatic count affected by lipemia, pathologist review confirmed adequate number.

**Table 3:** Serum biochemistry values in a dog presented for lamotrigine toxicity

Parameter	Concentration Day 1	Concentration Day 2	Concentration Day 3	Reference interval
Glucose (mg/dL)	87	143	111	63‐114
Creatinine (mg/dL)	2.1	0.8	0.9	0.5‐1.5
BUN (mg/dL)	24	7	10	9‐31
Phosphorus (mg/dL)	6.4	3	4.1	2.5‐6.1
Calcium (mg/dL)	8	9.4	9.1	8.4‐11.8
Sodium (mmol/L)	140	142	144	142‐152
Potassium (mmol/L)	4.1	3.9	4.3	4‐5
TCO_2_ (mmol/L)	11	22	23	13‐27
Total protein (g/dL)	3.7	5	5.1	5.5‐7.5
Albumin (g/dL)	2.1	3.1	2.8	2.7‐3.9
Globulin (g/dL)	1.6	1.9	2.3	2.4‐4
ALT (U/L)	571	1287	1033	18‐121
AST (U/L)	321	831	245	16‐55
ALKP (U/L)	122	381	1001	5‐160
GGT (U/L)	11	7	27 U/L	0‐13
Bilirubin—Total (mg/dL)	0.5	1.1	1.6	0‐0.3
Cholesterol (mg/dL)	110	189	170	131‐345
Triglyceride (mg/dL)	8852	1255	67	20‐150
Creatinine Kinase (U/L)	435	3987	1266	10‐200

On Day 2 two the patient was quiet, alert and responsive. Normal sinus rhythm was observed throughout the day (HR 109‐148 bpm). Respiratory rate and effort remained within normal limits. Thoracic auscultation was unremarkable, with the exception of referred upper airway noise. Systolic blood pressures ranged from 98‐128 mm Hg (range 100‐160 mm Hg). The dextrose was discontinued and the patient remained normoglycemic (84‐107 mg/dL; range 70‐130 mg/dL). Oxygen saturation remained normal without the need for oxygen supplementation. Neurologic examination was within normal limits. A repeat CBC (Idexx Reference Laboratory) revealed persistent polycythemia, and a mild inflammatory leukogram (Table 2). Repeat chemistry (Idexx Reference Laboratory) profile included further elevations in liver enzymes, triglycerides, and CK (Table 3). Intravenous fluid therapy (LRS; Dechra Veterinary Products + 20 meq KCl; Hospira Inc/L) was continued at a rate of 100 mL/h IV. Adjunctive treatments included continuation of maropitant citrate (Zoetis Inc.; 1 mg/kg IV SID), pantoprazole (NeuroMedics Pharma LLC; 1 mg/kg IV SID), metoclopramide (Metoclopramide Hydrochloride; 2 mg/kg/day IV), enrofloxacin (Bayer HealthCare LLC; 15 mg/kg IV SID) and unasyn (MITIM S.R.L; 30 mg/kg IV TID).

On Day 3 the patient was bright, alert and responsive. Normal sinus rhythm was observed throughout the day (HR 104‐132 bpm). Respiratory rate and effort remained within normal limits. Thoracic auscultation was unremarkable, with the exception of previously noted referred upper airway noise. Normotension (SBP 110‐128 mm Hg; range 100‐160 mm Hg), and normoglycemic (100‐117 mg/dL; range 70‐130 mg/dL) were observed. A repeat chemistry (Idexx Reference Laboratory) panel revealed mild improvements in ALT and AST, and further increases in ALKP, GGT, and bilirubin (Table 3). Repeat thoracic radiographs showed resolution of the previously noted increased opacity in the left cranial lung field, and a faint area of increased opacity superimposing the right middle lung lobe region. This finding was considered to be residual atelectasis or pneumonia. A mild interstitial pattern remained throughout the pulmonary parenchyma.

The patient was transitioned onto oral medications, and discharged home with cerenia (Maropitant citrate tablets, Zoetis Inc., Kalamazoo, MI; 48 mg PO SID), enrofloxacin (Enrofloxacin tablets, Bayer HealthCare LLC, Animal Health Division, Shawnee Mission, KS; 272 mg PO SID), clavamox (Amoxicillin trihydrate/clavulanate potassium tablets, Zoetis Inc., Kalamazoo, MI; 312.5 mg PO BID), metoclopramide (Metoclopramide 5 mg/5 mL Pharmaceutical Associates Inc, Greenville, SC; 7.5 mg PO TID), ursodiol (Ursodiol tablets, Par Formulations Private Limited, Kanchipuram, Tamil Nadu, India; 250 mg PO SID), and omeprazole (Omeprazole tablets, Glenmark Pharmaceuticals Inc., Mahwah, NJ; 20 mg PO SID). The recommendation was made to pursue an echocardiogram in the near future to further assess the heart; however, the patient was lost to follow‐up.

## 3 DISCUSSION

This case report describes severe cardiotoxicity secondary to LTG ingestion in a dog. Lamotrigine is known to cause intraventricular conduction delays due to its activity as a sodium channel blocker. A 2016 review of published human case reports identified cardiac toxic effects including left and right bundle branch blocks, prolongation of QT intervals, wide complex tachycardia (WCT), and most commonly sinus tachycardia. Complete heart block and pulseless VT were also described.^1^ In a 2018 review of 51 cases of acute LTG overdose, the most frequently reported cardiac abnormality was tachycardia; with 21% of people demonstrating QRS widening, and 6% of patients experiencing cardiac arrest.^10^ Doses of 100‐40 000 mg have been reported to cause CNS and cardiac toxicity in people.^1^ The time between toxic ingestion and presentation to the emergency room is frequently unknown. As such, measured levels of LTG may not correspond to the severity of clinical signs^10^ and drug levels are not clinically relevant in the acute setting due to the time delay in obtaining results.^9^ Levels of LTG were not measured in this case report.

There are limited reports of LTG toxicity in the veterinary literature. A review of the ASPCA APCC database from 2003‐2011 identified LTG exposure in 128 dogs and 10 cats. Eight animals (6%) are reported to have died due to the exposure.^2^ The reported lethal doses in mice and rats are 245 mg/kg and 205 mg/kg respectively. In dogs, doses of 3.4 mg/kg have been reported to induce lethargy and somnolence. Cardiac signs have been seen at doses over 20 mg/kg. Doses of >40 mg/kg have been reported to cause life‐threatening arrhythmias and seizures. Cats may be more sensitive due to their low capacity for glucuronidation; a cat was reported to develop bradycardia and ventricular premature complexes after ingesting a dose of 5 mg/kg^2^. Of the reported canine deaths, refractory seizures and sudden cardiac arrest, likely secondary to arrhythmias, were reported. One dog; a 1.5‐year‐old healthy Labrador retriever ingested 67.8 mg/kg of LTG and suffered VT, VPC's, and bundle branch block before cardiac arrest.^11^ A case report of a 7 month old male Labrador who ingested 278 mg/kg LTG in the extended release form describes vomiting, dull mentation, vertical nystagmus, extensor limb rigidity, and multifocal ventricular tachycardia.^5^ Treatment with single doses of methocarbamol and lidocaine, in conjunction with IVF, led to complete resolution of symptoms. Residual VPCs were noted 3 hours later, and NSR was observed 24 hours after presentation.^5^ Given the dose‐dependent cardiotoxicity of LTG and the massive dose ingested, it is remarkable that that patient survived. The patient in the current case report ingested a lower dose of LTG (26 mg/kg) and suffered severe tachyarrhythmias. The authors observed increases in LES on blood taken shortly after arrival. No pre‐existing blood work was available for this patient. Given the hepatic metabolism of LTG, it is feasible that pre‐existing liver dysfunction could have contributed to the severity of toxic effects in the case reported here. That said, increases in LES (ALT, AST, GGT, ALT, and total bilirubin) have been reported as rare side effects of LTG according to the FDA data information sheet for the medication.^3^ Increases in LES may also be caused by decreased perfusion to the liver secondary to decreased cardiac output. The presence of pancreatitis could also not be ruled out. An intermittent accelerated idioventricular rhythm, and VPC's continued in the patient reported here for 24 hours; which is similar to the duration of VPC's noted in the aforementioned case report.^5^ It is possible that myocardial damage was sustained during the period of hypotension and hypoperfusion leading to residual VPC's. A pre‐existing cardiac arrhythmia in our patient cannot be ruled out due to incomplete medical history, however, is unlikely given the complete resolution of VPC's >24 hours after treatment.

Treatment of LTG toxicity is based upon clinical signs. General treatment considerations for toxicities include decontamination via induction of emesis, gastric lavage, and administration of activated charcoal to reduce toxin absorption. The patient in this report had already vomited multiple times prior to presentation, and aspiration pneumonia was documented at the time of presentation, therefore induction of emesis was considered contraindicated. Given the time delay between ingestion and presentation, and the cardiovascular instability exhibited by the patient, anesthesia for gastric lavage was not recommended. Activated charcoal requires oral administration, and due to the altered mental state of the patient this was not recommended either.

The most life‐threatening symptom exhibited by the patient in this report was the arrhythmia. Lamotrigine toxicity causes a delay in intraventricular conduction, resulting in prolonged PR intervals, QRS complexes and QT intervals, and AV block. Prolongation of the QT interval increases the risk for ventricular arrhythmias, torsade de point, and sudden death.^2^ The patient in this report was administered lidocaine; initially with a positive effect, demonstrated by conversion to NSR. Lidocaine is a class Ia sodium channel blocker; and is the first line of treatment for VT. It has been used to treat toxicity induced by other sodium channel blockers with the hypothesis that lidocaine's fast kinetics with sodium channel binding reduce the fraction of blocked sodium channels when used with a sodium channel blocker with slower kinetics.^12^ The VT exhibited by this patient persisted in spite of an increasing dose of lidocaine, and as such, other treatments were sought.

Alkalization via administration of sodium bicarbonate (NaHCO_3_) has been recommended in human cases of LTG when cardiotoxicity is suspected secondary to sodium channel blockade. The exact mechanism by which NaHCO_3_ acts to reverse sodium channel blockade is not fully understood. It is possible that the increase in sodium concentration, alteration in pH, or a combination thereof reverses the sodium channel blockade. This is evidenced by experimental studies in dogs evaluating the effect of NaHCO_3_ on conduction in cases of cocaine and amitryptiline toxicity, and flecainide and mexiletine‐treated purkinje fibers.^12^ Twenty percent of patients in the 2018 review of LTG overdose were given NaHCO_3_ as part of their treatment, and the effect was variable. The QRS complex did not correct in four cases.^10^ Detrimental side effects of sodium bicarbonate are well documented and include acute alterations in sodium concentration, hypokalemia, and paradoxical acidosis.^13^ Patients administered NaHCO_3_ must have the ability to ventilate appropriately as a by‐product of metabolism in vivo is CO_2_. Given that the patient reported here was brachycephalic, had aspiration pneumonia, and weakness caused by decreased cardiac output, small doses of sodium bicarbonate were administered with caution. Fortunately, the patient's venous partial pressure of CO_2_ was not negatively affected by the sodium bicarbonate; however, no discernible response on the rhythm disturbance was observed.

Intralipid emulsion therapy was administered in six human cases reported by Alhaya et al. Two of those patients had ingested LTG only, and administration of ILE rapidly narrowed the QRS complex and resolved the conduction delay.^10^ Chavez et al state that a potential role for ILE has been described in patients with toxic levels of LTG and ECG changes refractory to treatment with bicarbonate. They describe immediate resolution of ECG changes upon administration of ILE to a man suffering LTG toxicity that had failed bicarbonate therapy.^7^ Lipid emulsion was described as a rescue therapy in LTG overdose by Castanares‐Zapatero et al, who reported a case of a 50‐year‐old woman that suffered ventricular arrhythmias refractory to treatment with NaHCO_3_ and magnesium sulfate. That patient demonstrated normalization of conduction disturbances almost immediately upon administration of ILE.^8^ Nogar et al describe the use of ILE in a 48‐year‐old woman suffering cardiovascular collapse, widening of the QRS complex in spite of bicarbonate therapy, and subsequent pulselessness—requiring cardiopulmonary resuscitation. Lipid therapy was administered as a “rescue” in this case, and the authors comment that in retrospect, higher doses of ILE or a CRI of ILE could have been considered.^9^ The exact mechanism by which ILE is useful in toxicities is unclear; however, a number of theories have been proposed. It is possible that lipid emulsion may act as a “lipid sink,” sequestering lipophilic compounds and preventing them from reaching their site of action. The metabolic theory proposes that increasing the serum concentration of free fatty acids (FFA's) via ILE infusion results in increased FFA uptake by myocardial cells, providing substrates for beta‐oxidation and ATP production, which can lessen the cardiotoxic effects of certain drugs. Potential adverse effects of ILE include pancreatitis due to persistent lipemia, hypersensitivity reactions, fat overload syndrome, and lipid emboli, as well as infection if the product is contaminated. Lipemia was observed in the patient reported here; as were increases in LES, which could have been in part attributed to pancreatitis. However, no clinical deleterious side effects occurred. An important consideration when using ILE is interference of drugs administered for supportive care.^5^ In this case report, lidocaine was used to treat the ventricular arrhythmias. Lidocaine is a local anesthetic and a class of drug for which systemic toxicity is commonly treated with ILE.^6^ Additional doses of lidocaine were administered with positive effect in the early part of ILE therapy in our patient; however, repeat doses were needed, likely due to enhanced clearance of lidocaine by ILE.

This case report is the first in the veterinary literature to document the successful use of ILE for the treatment of severe LTG cardiotoxicity in a dog; a life‐saving treatment for this patient. As a result, the authors suggest that ILE therapy should be considered as an early treatment intervention if cardiotoxicity is observed secondary to LTG ingestion in dogs.

## CONFLICT OF INTEREST

None declared.

## AUTHORSHIP

TJB: wrote the discussion and contributed to the case summary. LG: wrote the case summary and contributed to the discussion
